# Upregulation of the Antioxidant Response-Related microRNAs miR-146a-5p and miR-21-5p in Gestational Diabetes: An Analysis of Matched Samples of Extracellular Vesicles and PBMCs

**DOI:** 10.3390/ijms26146902

**Published:** 2025-07-18

**Authors:** Jovana Stevanović, Ninoslav Mitić, Ana Penezić, Ognjen Radojičić, Daniela Ardalić, Milica Mandić, Vesna Mandić-Marković, Željko Miković, Miloš Brkušanin, Olgica Nedić, Zorana Dobrijević

**Affiliations:** 1 Institute for the Application of Nuclear Energy, University of Belgrade, 11080 Belgrade, Serbia; jovana.stevanovic@inep.co.rs (J.S.); ninoslavm@inep.co.rs (N.M.); anap@inep.co.rs (A.P.); olgica@inep.co.rs (O.N.); 2 Clinic for Gynecology and Obstetrics “Narodni Front”, 11000 Belgrade, Serbia; ogi.radojicic@gmail.com (O.R.); ardalic.daniela@gakfront.org (D.A.); milica96mandic@gmail.com (M.M.); vesna.mandic-markovic@med.bg.ac.rs (V.M.-M.); zeljko.mikovic@med.bg.ac.rs (Ž.M.); 3 Medical School, University of Belgrade, 11000 Belgrade, Serbia; 4 Centre for Human Molecular Genetics, Faculty of Biology, University of Belgrade, 11000 Belgrade, Serbia; milosb@bio.bg.ac.rs

**Keywords:** miR-21-5p, miR-155-5p, miR-146a-5p, oxidative stress, redox status, gestational diabetes, iron, superoxide dismutase, adverse outcomes

## Abstract

MicroRNA-based regulatory mechanisms show disturbances related to oxidative stress (OS) interconnected with inflammation (IFM), as well as impairments associated with gestational diabetes (GDM). The aim of this study was to assess the diagnostic and prognostic significance of the OS/IFM-related microRNA in GDM by using peripheral blood mononuclear cells (PBMCs) and serum-derived extracellular vesicles (EVs) as biological samples. We selected the known OS/IFM-associated microRNAs miR-146a-5p, miR-155-5p, and miR-21-5p as candidates for our GDM biomarker analysis. Quantitative RT-PCR was employed for relative quantification of the selected microRNAs from paired samples of PBMCs and EVs derived from patients with GDM and healthy controls (*n* = 50 per group). The expression levels were analyzed for correlations with lipid and glycemic status indicators; metal ion-related parameters; serum thiol content; protein carbonyl and thiobarbituric acid-reactive substances’ (TBARS) levels; glutathione reductase (GR), Superoxide dismutase (SOD), and catalase (CAT) activity; and NRF2 expression. MiR-146a-5p and miR-21-5p were significantly upregulated in both PBMCs and EVs obtained from GDM patients. EVs-miR-21-5p showed a positive correlation with glycemic status in GDM patients, while miR-155-5p from PBMCs demonstrated correlation with iron-related parameters. The expression of selected microRNAs was found to correlate with NRF2 expression and SOD activity. The level of miR-146a-5p negatively correlated with neonatal anthropometric characteristics, while a higher level of PBMCs-miR-21-5p expression was determined in GDM patients with adverse pregnancy outcomes (*p* = 0.012). Our data demonstrate a disturbance of OS/IFM-microRNAs in GDM and illustrate their potential to serve as indicators of the associated OS-related changes, neonatal characteristics, and adverse pregnancy outcomes.

## 1. Introduction

Gestational diabetes mellitus (GDM) is the most commonly diagnosed metabolic disorder in pregnancy, affecting around 12% of pregnant women in European countries, as estimated by the International Diabetes Federation (IDF) [1]. In addition to its high prevalence, concerning statistics that link the occurrence of GDM with the development of severe obstetric and neonatal complications qualify this disorder as one of the major pregnancy-related health issues. Imminent complications related to GDM may cause direct severe adverse effects on pregnancy progression, as well as detrimental health outcomes for both the neonate and the mother. Furthermore, the long-term consequences of diabetic pregnancies include an increased susceptibility to metabolic disorders, such as Type 2 diabetes (T2DM), in both affected women and their offspring [2].

Since GDM is diagnosed relatively late in pregnancy, after the onset of damaging mechanisms, there has been a growing effort among researchers and clinicians to identify efficient molecular markers that could facilitate early risk stratification and identification of affected women [3]. In addition to their diagnostic potential, the prognostic significance of molecular markers in terms of the development of severe pregnancy complications of GDM has become a focus of interest, particularly given the limited applicability of conventional biochemical parameters related to glucose and lipid metabolism. As in other multifactorial human diseases, microRNAs have emerged as potential plausible candidates for such research due to their desirable chemical properties, abundance, and regulatory roles [4,5].

MicroRNAs play a crucial regulatory role in different cellular processes involved in the molecular basis of GDM, pregnancy maintenance, placentation, fetal nutrition, and severe pregnancy complications associated with hyperglycemia [6,7,8,9,10]. Additionally, various microRNA species have been shown to influence antioxidant defense mechanisms and immune-system regulation [11,12]. Consequently, their dysregulation may contribute to the development of GDM-associated complications, since oxidative stress (OS) and inflammation (IFM) are interconnected, associated with hyperglycemia, and recognized as major hazardous contributors to the development of GDM-related conditions [13,14]. This constellation of OS/IFM-governed pregnancy complications linked to hyperglycemia includes such severe health disturbances as gestational hypertension, preeclampsia, and amniotic fluid and fetal growth disorders [2]. Therefore, OS/IFM-related microRNAs hold promise as potential biomarkers for GDM diagnosis, prediction of adverse pregnancy outcomes, and monitoring of metabolic status and treatment efficacy in hyperglycemic pregnancies.

The most promising microRNA-based biomarkers for clinical usage are typically assessed through liquid biopsy analyses, including those that examine the cargo of extracellular vesicles (EVs) derived from tissues primarily affected by elevated glucose and reactive oxygen species (ROS) in the bloodstream [15]. In the context of impaired glucose tolerance, increased ROS production, exacerbated oxidative stress, and inflammation, microRNAs from peripheral blood mononuclear cells (PBMCs) have gained considerable attention in biomarker research on diabetic states [16,17,18]. These cells are extremely responsive to oxidative stress, act as effector cells in the immune response, and produce cytokines which modulate inflammatory pathways [19,20]. Despite this potential, the available data on GDM-associated microRNAs from PBMCs are scarce, while studies focusing on plasma and serum have produced inconsistent and sometimes contradictory findings [18,21]. These discrepancies may be attributed to variations in study design, patient recruitment criteria, and various preanalytical factors that influenced the microRNA profiling results [5,22]. Furthermore, microRNA profiles show pregnancy-stage-related fluctuations, which makes gestational age a confounding factor that directly influences microRNA quantification results [23]. Therefore, the selection of an appropriate biological sample for the quantification of GDM-related microRNAs and the timing of sample collection (relevant gestational stage) are critical for generating reliable and reproducible results in GDM biomarker research. To address this, the present study employed matched samples of PBMCs and extracellular vesicles. In our case-control study of GDM, we focused on three microRNAs with known OS/IFM-related functions: miR-146a-5p, miR-155-5p, and miR-21-5p [24,25,26,27,28,29,30,31]. The selection of candidate microRNAs for this study was based on their experimentally confirmed functional properties, specifically their involvement in OS/IFM-related processes. All three selected microRNAs—miR-146a-5p, miR-155-5p, and miR-21-5p—are relatively abundant in blood compartments, while their OS/IFM-related activities were previously confirmed in tissues severely affected by hyperglycemia [24,25,26,27,28,29,30,31]. Their association with pregnancy pathologies was demonstrated by microRNA profiling of placental tissues, as well as by targeted microRNA quantification, functional characterization, and mechanistic studies in extraembryonic tissues and the corresponding cell lines [32,33,34,35,36,37,38,39,40,41,42,43,44]. Furthermore, the biomarker properties of these microRNAs have been previously detected in other diabetic states, as well as in disorders associated with immune-system dysregulation [12,45].

We sought to evaluate the changes in the expression of miR-146a-5p, miR-155-5p, and miR-21-5p between GDM patients and healthy controls. To compare different biological sources of microRNAs with potential biomarker value in GDM and to explore the diagnostic potential of selected microRNAs, we quantified the chosen microRNAs in PBMCs and paired samples of serum-derived EVs. Given the limited data on microRNA expression in PBMCs of GDM patients [18], the evaluation of a microRNA panel may aid in clarifying the role of microRNAs in metabolic dysregulation, OS, and IFM associated with GDM, while also evaluating their biomarker potential. Another objective of this study was to evaluate the prognostic significance of the selected microRNA panel with respect to neonatal characteristics and the occurrence of adverse pregnancy outcomes. Although some of the selected microRNAs have been previously investigated as potential GDM biomarkers, the use of relatively neglected biological sources of microRNAs, the comparison of paired samples of biological material originating from the same individuals, and the testing for associations with pregnancy complications represent the basis for acquiring valuable novel findings. We also investigated the potential of OS/IFM-related microRNAs—miR-146a-5p, miR-155-5p, and miR-21-5p—as indicators of OS in GDM by correlating their expression with the activity of antioxidant enzymes in the circulation, NRF2 expression, and levels of several markers of oxidative damage of biomacromolecules. These results would provide an invaluable basis for designing larger replication studies, which could result in the identification and characterization of microRNA biomarkers relevant to monitoring the response to antioxidative interventions in GDM. Furthermore, we evaluated the relevance of these microRNAs in assessing the metabolic state of GDM patients through correlation analyses involving lipid and glucose status parameters.

## 2. Results

The basic characteristics of GDM patients and controls, as detailed in our previous article [46] and presented in Supplementary Table S1, indicate that there were no statistically significant differences in age or gestational week at the time of sampling between the two groups.

**Figure 1:**
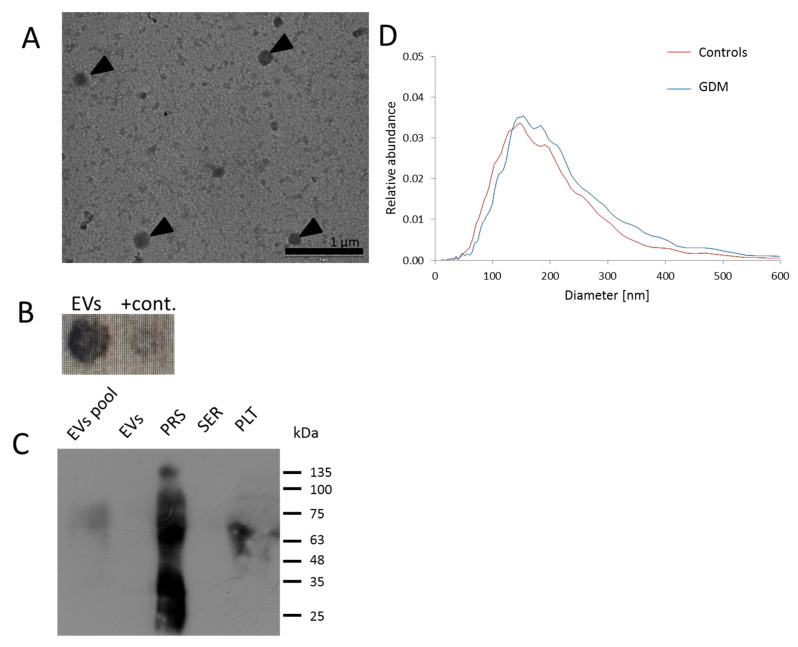
Characterization of isolated serum-derived EVs. (**A**) TEM analysis of EV isolates; arrowheads point to round-shaped EVs. (**B**) CD63 positivity of pooled EV isolates (~10^11^/mL) determined by dot blot analysis; +contr.—positive control (prostasomes). (**C**) Immunoblot with anti-CD63 antibody, confirming the presence of CD63 in pooled EVs (~10^11^/mL) and positive controls (PRS—prostasomes and PLT—platelets); signal was not detected for lower EV concentrations (EVs, ~10^9^/mL) and serum sample (SER). (**D**) NTA analysis of EVs derived from samples corresponding to GDM patients and the control group; size distribution plot of isolated EVs, with *x*-axis representing the diameter of detected particles in nm, and *y*-axis indicating the relative abundance of the detected particles.

To confirm the presence of EVs in the isolates and assess the yield of the isolation procedure, pooled samples from study participants were subjected to transmission electron microscopy (TEM) analysis, nanoparticle tracking analysis (NTA), and immunoblotting. TEM analysis revealed the presence of round structures that correspond to the size and morphology characteristics of serum EVs (Figure 1A). Dot blot (Figure 1B) and Western blot (Figure 1C) analysis confirmed the presence of the EV marker CD63 in the isolates. NTA analysis provided additional evidence of EVs, demonstrating particle sizes ranging from approximately 40 to 600 nm, with the majority falling between 100 and 300 nm in diameter (Figure 1D). NTA in scattering mode indicated no statistically significant differences in the concentration of EVs isolated from randomly selected samples obtained from GDM patients and controls (*p* = 0.47). The median sizes of EVs in both groups were comparable (*p* = 0.318), and the difference in size values corresponding to the 10th and 90th percentiles was not significant (*p* = 0.452 and *p* = 0.531, respectively).

**Figure 2:**
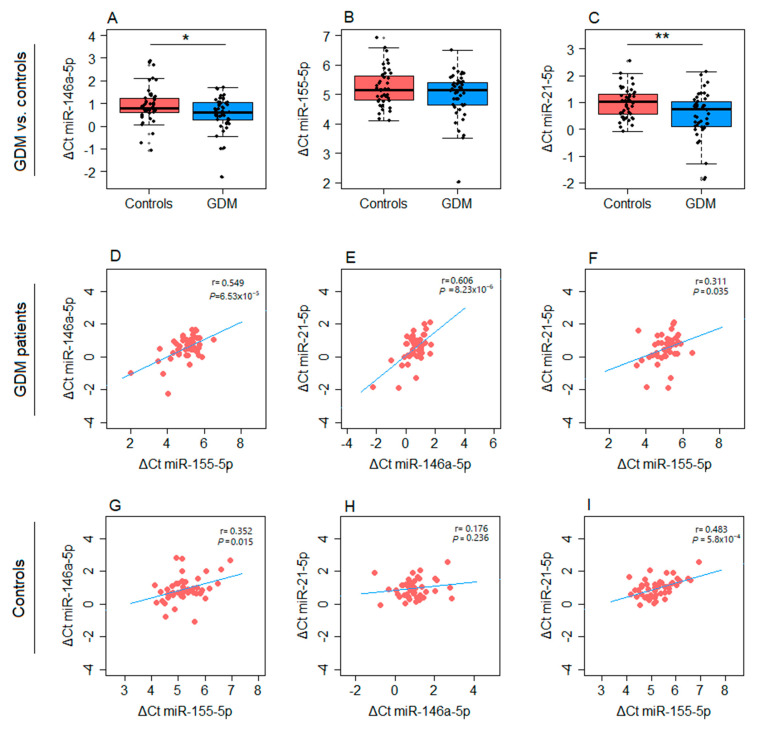
Differences in the expression of microRNAs from PBMCs between GDM patients and controls: (**A**) miR-146a-5p, (**B**) miR-155-5p, and (**C**) miR-21-5p. Correlation between the expression of PBMCs-derived microRNAs in GDM patients: (**D**) miR-146a-5p and miR-155-5p, (**E**) miR-146a-5p and miR-21-5p, and (**F**) miR-155-5p and miR-21-5p. Correlation between the expression of PBMC-derived microRNAs in controls: (**G**) miR-146a-5p and miR-155-5p, (**H**) miR-146a-5p and miR-21-5p, and (**I**) miR-155-5p and miR-21-5p. Box plots are used for depicting differences in the expression of microRNAs between groups, with boxes representing medians with interquartile ranges and whiskers indicating min and max values; Student’s *t*-test was employed for analyzing statistical significance; *p* < 0.05 and *p* < 0.01 are indicated by one and two asterisks, respectively. Scatter diagrams are used for depicting the relationship between the expression levels of pairs of microRNAs, with Pearson’s correlation coefficient (r) and the corresponding *p*-value from the linear regression analysis presented within each diagram.

Isolated EVs were used to analyze the expression of miR-146a-5p and miR-21-5p, which were also evaluated in paired samples of PBMCs obtained from GDM patients and controls. MiR-155-5p, however, was reliably quantified only in the PBMCs (Figure 2). Both miR-146a-5p and miR-21-5p were significantly upregulated in the PBMCs of GDM patients (*p* = 0.020 and *p* = 0.004, respectively) (Figure 2A,C). In contrast, the difference in miR-155-5p expression between cases and controls was not statistically significant (*p* = 0.067) (Figure 2B). All three tested microRNAs from PBMCs showed positively correlated expression with each other in GDM patients (Figure 2D–F), with the strongest correlation observed for miR-146a-5p and miR-21-5p (r = 0.606, *p* < 0.001) (Figure 2E). Conversely, no correlation was detected between these two microRNAs in the control group; however, a significant correlation between miR-155-5p and both miR-146a-5p and miR-21-5p was observed (Figure 2G–I).

**Figure 3:**
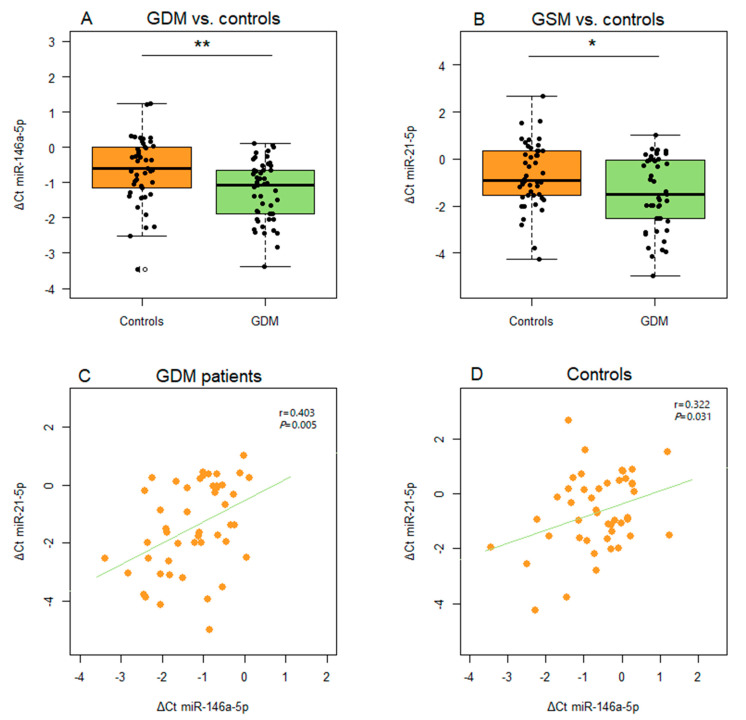
Differences in the expression of microRNAs from EVs between GDM patients and controls: (**A**) miR-146a-5p and (**B**) miR-21-5p. Correlation between the expression of EV-derived miR-146a-5p and miR-21-5p in (**C**) GDM patients and (**D**) controls. Box plots are used for depicting differences in the expression of microRNAs between groups, with boxes representing medians with interquartile ranges and whiskers indicating min and max values; Student’s *t*-test was employed for analyzing statistical significance; *p* < 0.05 and *p* < 0.01 are indicated by one and two asterisks, respectively. Scatter diagrams are used for depicting the relationship between the expression levels of pairs of microRNAs, with Pearson’s correlation coefficient (r) and the corresponding *p*-value from the linear regression analysis presented within each diagram.

**Figure 4:**
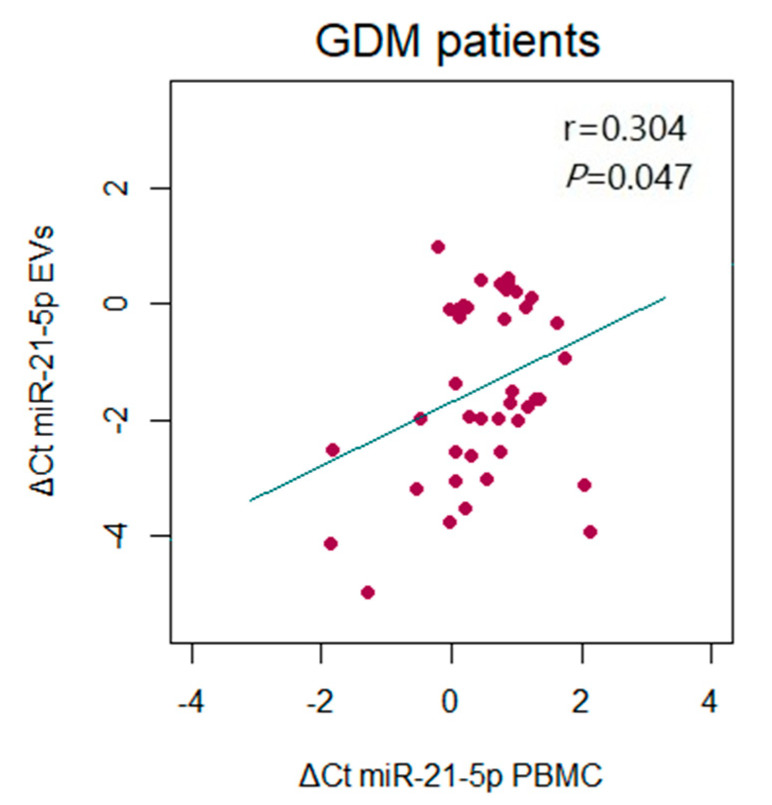
Correlation between the expression of miR-21-5p from PBMCs and serum-derived EVs in GDM patients. Scatter diagrams are used for depicting the relationship between the expression levels of miR-21-5p in sample pairs, with Pearson’s correlation coefficient (r) and the corresponding *p*-value from the linear regression analysis presented within the diagram.

Similar findings were obtained for microRNA expression in serum-derived EVs, with upregulaton of miR-146a-5p and miR-21-5p associated with a GDM diagnosis (*p* = 0.002 and *p* = 0.014, respectively) (Figure 3A,B). Furthermore, a statistically significant positive correlation was detected between the levels of miR-146a-5p and miR-21-5p in EVs in both GDM patients and controls (r = 0.403, *p* = 0.005 and r = 0.322, *p* = 0.031, respectively) (Figure 3C,D). When the expression of the analyzed microRNAs in paired samples of PBMCs and EVs was compared, a marginally significant correlation was observed between the expression levels of miR-21-5p in these two biological samples in the GDM patient group (Figure 4).

The expression levels of the analyzed microRNAs in PBMCs and EVs did not show any correlation with pre-pregnancy body mass index (BMI) or BMI at the time of sampling in either GDM patients or controls. When analyzing the potential correlations between the expression of selected microRNAs with lipid and glycemic status indicators in GDM, only miR-21-5p showed a mild correlation with OGT testing results. All other correlation analyses yielded statistically insignificant results (Table 1).

**Figure 5:**
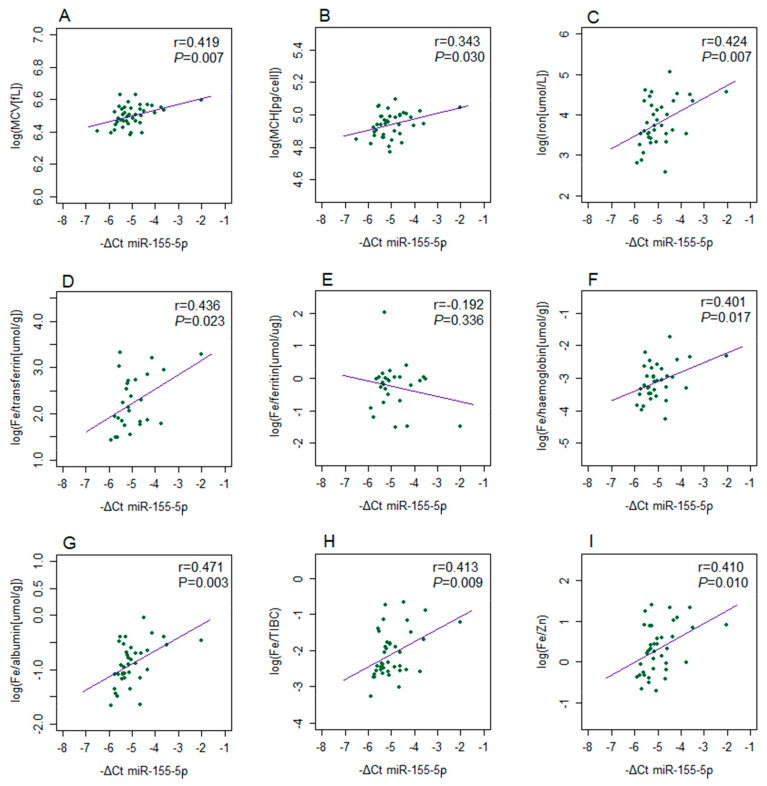
Correlations between the expression of miR-155-5p in PBMCs (−ΔCt) and the values (log2n) of iron-related parameters in GDM patients: (**A**) MCV, (**B**) MCH, (**C**) serum iron concentration and ratios between iron concentration and (**D**) transferrin, (**E**) ferritin, (**F**) hemoglobin, (**G**) albumin, (**H**) total iron binding capacity (TIBC), or (**I**) serum zinc concentrations. Pearson’s correlation coefficient (r) and the corresponding *p*-value from the linear regression analysis are presented within each diagram.

Blood cell count parameters did not show statistically significant correlation with the expression levels of the tested microRNAs in either PBMCs or EVs from GDM patients with the exception of miR-155-5p. Specifically, a significant positive correlation was observed between miR-155-5p expression in PBMCs and both mean corpuscular volume (MCV) and Mean corpuscular hemoglobin (MCH) values (Figure 5A,B). The direction of these correlations was consistent with the positive association between miR-155-5p expression and the iron level in GDM patients (r = 0.424, *p* = 0.007) (Figure 5C). Similar correlations were observed for the ratios of iron level and concentrations of several metal iron carrier proteins from plasma, as well as for the iron-to-zinc ratio (Figure 5). No significant correlations were found between the expression levels of other tested microRNAs and metal ion-related parameters in GDM.

Regarding biochemical parameters indicative of acute inflammation, no significant correlations were observed between the expression levels of the analyzed microRNAs and C-reactive protein (CRP), aspartate aminotransferase (AST), or alanine aminotransferase (ALT) values. However, in GDM patients, the expressions of miR-146a-5p and miR-155-5p in PBMCs were significantly negatively correlated with fibrinogen levels (r = −0.398, *p* = 0.018 and r = −0.387, *p* = 0.022, respectively).

The expressions of miR-155-5p and miR-21-5p in PBMCs demonstrated significant positive correlations with NRF2 protein levels (r = 0.401, *p* = 0.042 and r = 0.396, *p* = 0.050, respectively), while miR-21-5p also demonstrated a positive correlation with the SOD activity in circulation (r = 0.361 and *p* = 0.024) (Table 2). Such correlation between miR-21-5p expression and redox status-related parameters was not detected in EV samples. In contrast, the expression of miR-146a-5p in EVs was inversely correlated with both *NFE2L2* expression (encoding NRF2) (r = −0.357, *p* = 0.012) and SOD activity (r = −0.400, *p* = 0.011) in GDM patients. No statistically significant correlations were found between PBMCs-derived miR-146a-5p and redox status-related parameters (Table 2). Data on the indicators of antioxidant response and redox status in GDM patients included in the present study are presented in Stevanović et al. [46].

**Figure 6:**
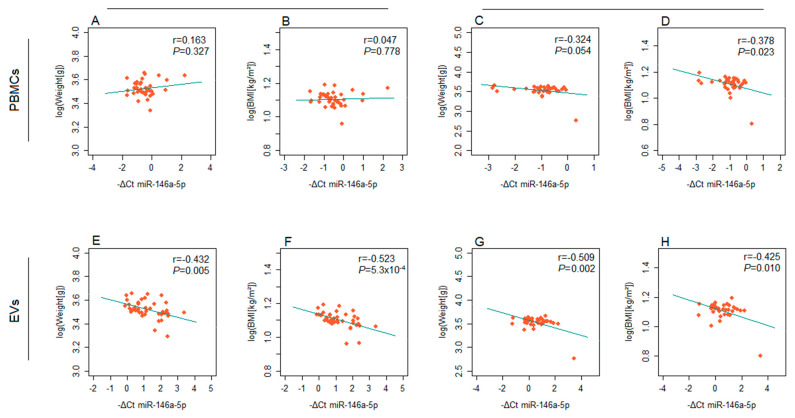
Correlations between the expression of miR-146a-5p (−ΔCt) and the values (log2n) of anthropometric characteristics of neonates: miR-146a-5p from PBMCs of GDM patients and (**A**) neonatal weight and (**B**) BMI; miR-146a-5p from PBMCs of the control group and (**C**) neonatal weight and (**D**) BMI; miR-146a-5p from EVs of GDM patients and (**E**) neonatal weight and (**F**) BMI; miR-146a-5p from EVs of the control group and (**G**) neonatal weight and (**H**) BMI. Pearson’s correlation coefficient (r) and the corresponding *p*-value from the linear regression analysis are presented within each diagram.

**Figure 7:**
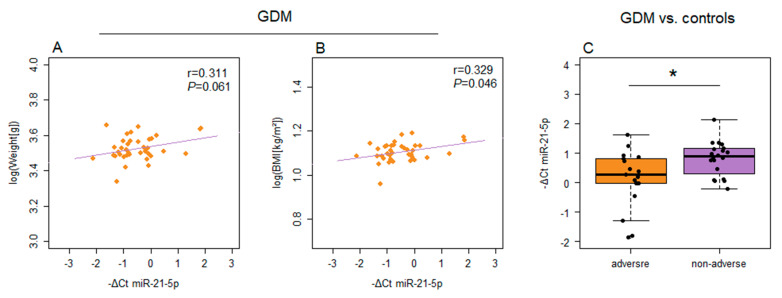
Correlations between the expression of PBMCs-derived miR-21-5p and anthropometric characteristics of the neonates: (**A**) weight and (**B**) BMI. (**C**) Differences in the expression of miR-21-5p from PBMCs between GDM patients with and without adverse pregnancy outcomes. Scatter diagrams are used for depicting the relationship between the expression level of miR-21-5p and the values of anthropometric characteristics, with Pearson’s correlation coefficient (r) and the corresponding *p*-value from the linear regression analysis presented within each diagram. Box plots are used for depicting differences in the expression of miR-21-5p between groups, with boxes representing medians with interquartile ranges and whiskers indicating min and max values; Student’s *t*-test was employed for analyzing statistical significance; *p <* 0.05 is indicated by an asterisk.

Both miR-146a-5p and miR-21-5p showed correlation between their expression level and the values of neonatal anthropometric characteristics (Figure 6 and Figure 7). Specifically, miR-146a-5p expression in EVs was inversely correlated with newborn weight and BMI in both GDM patients and controls. Similarly, miR-146a-5p expression in PBMCs showed a significant negative correlation with these parameters in the control group (Figure 6). As for miR-21-5p, the correlation coefficients indicated a direct correlation of the expression level in PBMCs with the neonates’ birth weight and BMI in GDM patients (Figure 7A,B). However, the correlation with neonatal weight did not reach the threshold for statistical significance (Figure 7A). The expression of miR-21-5p in PBMCs significantly differed between patients with GDM when stratified according to pregnancy outcomes, with higher levels observed in the subgroup with adverse pregnancy outcomes (Figure 7C).

## 3. Discussion

Our findings demonstrate a significant upregulation of miR-146a-5p and miR-21-5p in GDM patients compared to normoglycemic controls in mid-to-late pregnancy stages. These results are consistent across both PBMC- and EV-derived microRNAs, which further augments the significance of the evaluated microRNAs as potential biomarkers of GDM, since the same direction of change in the expression of miR-146a-5p and miR-21-5p is noted in two different types of paired biological sources of microRNAs. However, a relatively weak correlation was found between the expression of microRNAs in PBMCs and EVs from the same participants, with this correlation being significant only for miR-21-5p. This suggests that the expression of microRNAs in PBMCs and other EV-producing cells in GDM is influenced by a complex regulatory network, while selective and tightly regulated microRNA sorting into EVs may have contributed to the observed variations in microRNA quantification results. Another similarity between the microRNA profiles derived from PBMCs and EVs is the positive correlation in the expression of selected microRNAs in both GDM patients and controls. These observations are in line with the hypothesis that selected microRNAs may be coregulated by similar or overlapping regulatory mechanisms in response to common stimuli present in both hyperglycemic and healthy pregnancies.

For miR-146a-5p, the observed direction of dysregulation in GDM is somewhat unexpected, since previous results indicated a downregulation of this microRNA in hyperglycemia and conditions accompanied by oxidative stress and inflammation, as well as in severe pregnancy pathologies [24,47,48]. Namely, high glucose conditions and ROS induction are associated with decreased expression of miR-146a-5p in different types of cultured cells [24,49], while circulatory levels of this microRNA are shown to be reduced in patients with T2DM and type 1 diabetes mellitus (T1DM) in most of the studies on this issue [47]. However, the expression of miR-146a-5p and its target genes involved in immune regulation are significantly increased in human umbilical vein endothelial cells (HUVECs) during the early phase of the hyperglycemic condition [50], which is consistent with our findings. This may imply that the effect could be disease- or tissue-specific, or that in later stages of GDM pregnancy, the results of miR-146a-5p quantification could resemble the results obtained in studies showing the downregulation of this microRNA. Additionally, the results of several studies on miR-146a-5p expression in the circulation in hyperglycemic conditions have shown an upregulation inconsistent with the generalized view of dysregulation in diabetic conditions [51,52]. Another study has also demonstrated gender differences in the expression of miR-146a-5p in T2DM vs. normoglycemic controls [53], which might have influenced the discordances between the findings related to T2DM and GDM. Apart from hyperglycemia, an important damaging component of diabetic conditions is inflammation, which contributes to changes in the expression of miR-146a-5p in the opposite direction, as demonstrated by an upregulation of this microRNA induced by proinflammatory cytokines [54].

As for previous reports on the biomarker properties of miR-146a-5p in GDM, this RNA has been identified as one of the top upregulated microRNAs in blood in a small number of studies [55,56]. Even though other studies have failed to confirm these findings, the microRNA profiling results of different blood fractions in GDM seem highly heterogeneous, and rare microRNAs have been reported as potential biomarkers in more than one study [57]. The direction of dysregulation in these previous reports matches our results and, therefore, supports our novel findings, even though other types of specimens for liquid biopsy were used [55,56].

As for miR-21-5p, which is another microRNA with a detected upregulation in our GDM group, previous findings have suggested an increase in expression in hyperglycemic conditions [58]. Its expression was induced in the islets of the murine model in T2DM and glucose-intolerant patients, while mechanistic studies have pointed out the role of miR-21-5p in the regulation of pancreatic β cell function in T2DM [59]. Furthermore, most of the studies on T2DM have demonstrated an elevated expression of the circulatory miR-21-5p, as well as its association with the development of diabetic complications [60]. Furthermore, this microRNA has been suggested as a biomarker of T2DM susceptibility in subjects with impaired glucose tolerance and an early predictor of ROS-mediated damage in subjects with a high risk of T2DM [61]. It has also been reported as a potential biomarker of T1DM, with an upregulation in EVs associated with inflammatory stimulation [62]. Our findings are in line with these previous observations concerning biomarker properties of miR-21-5p in diabetic conditions and the effect of hyperglycemia, ROS production, and proinflammatory cytokines on its expression. When it comes to studies on GDM, previous reports are conflicting. Namely, microRNA profiling and candidate gene expression analysis have rarely supported the dysregulation of miR-21-5p in GDM, while the passenger strand miR-21-3p has been reported as a potential GDM biomarker in several studies [63,64]. Guan et al. [65] have shown a GDM-related reduction in the expression of miR-21-5p in placentas, while in two other reports, this microRNA was found to be upregulated in serum samples from GDM patients and its expression correlated with glucose levels [66,67]. Disparities could be explained by the reported fluctuations in the expression of miR-21-5p during pregnancy progression, as well as by age-related differences, which were noted for placental expression of this microRNA and may reflect on the expression in other tissues affected by GDM [65,66]. Taking into account that our previous study on the same group of patients demonstrated a downregulation of lncRNA *MALAT1* in GDM, the observed upregulation of miR-21-5p was expected, since *MALAT1* acts as a microRNA sponge for miR-21-5p and the functional significance of this interaction has been previously demonstrated in the context of GDM [46,68]. Even though our present study is not mechanistic, the observed dysregulation is consistent with the existence of a *MALAT1*/miR-21-5p regulatory axis.

Both miR-146a-5p and miR-21-5p demonstrate poor significance as indicators of the metabolic status in GDM. PBMC-derived microRNAs fail to show any correlation with the values of lipid and glycemic status parameters. However, the expression of miR-21-5p in EVs positively correlates with glycemia in GDM patients, which is in line with previous findings and supports the role of the circulatory miR-21-5p as a glucose-tolerance indicator with potential significance in monitoring the efficiency of GDM treatment [67]. Our results are also compatible with the qualification of miR-21-5p as a glucose-sensing microRNA [31] and correspond with the findings on the enrichment of EVs with miR-21-5p in hyperglycemic conditions [69].

Although miR-155-5p was previously reported as dysregulated in the circulation of patients with GDM [4], our results do not support such findings. However, we did not manage to reliably quantify this microRNA in EVs, while the lack of diagnostic significance of PBMC-derived miR-155-5p in GDM is consistent with earlier reports focused on blood leukocytes or whole blood as microRNA sources [21,70]. Therefore, our findings do not identify miR-155-5p as a potential diagnostic biomarker of GDM in mid-to-late pregnancy stages. The expression of this microRNA demonstrates correlation with iron status-related parameters, which may be related to the strong connection between iron metabolism and inflammation, since miR-155-5p is highly responsive to inflammatory stimuli and is also a potent regulator of the inflammatory response [27,71]. This qualifies miR-155-5p as a potential indicator of iron metabolism in GDM patients, yet without diagnostic significance.

The results of the correlation analysis between the expression levels of microRNA and the values of redox status parameters identify PBMC-derived miR-155-5p and miR-21-5p as indicators of NRF2 protein level. The observed correlations may originate from the complex regulatory network of antioxidant response, since both miR-155-5p and miR-21-5p affect ROS production, which stimulates the stability and activation of NRF2 [29,31]. Furthermore, NRF2 was identified as a positive regulator of the expression of miR-21-5p precursor [72]. On the other hand, miR-155-5p and miR-21-5p are known as negative regulators of *NFE2L2*, while NRF2 may activate its own gene expression, which may partially explain the lack of correlation between the expression of microRNAs with the level of *NFE2L2* mRNA that encodes NRF2 [31,73,74]. A positive correlation between miR-21-5p expression and SOD activity is consistent with the upregulation of SOD genes by NRF2 activation in PBMCs.

For EV-derived microRNAs, correlation analyses revealed differences in the association with redox status parameters compared to the miR-146a-5p and miR-21-5p from PBMCs. EV-originating miR-21-5p failed to demonstrate a correlation with any of the analyzed indicators of the antioxidant response and redox status. On the other hand, the expression of EV-derived miR-146a-5p negatively correlates with *NFE2L2* mRNA level, as well as with SOD activity. This observation is expected, since the expression of miR-146a-5p is induced by ROS, while NRF2 is identified as a direct target of negative regulation by miR-146a-5p [75,76]. Additionally, *SOD2* encodes mRNA with a binding site for miR-146a-5p [75,77]. Taking into account the correlation coefficients, as well as the noted upregulation of miR-146a-5p and the reduced SOD activity in GDM [31], miR-146a-5p seems the most plausible candidate for a redox status indicator among the tested microRNAs.

EV-derived miR-146a-5p also shows improved characteristics as a predictor of neonatal weight and BMI compared to microRNA isolated from PBMCs. All the acquired correlation coefficients for the GDM group and for the controls were negative, in the range of 0.425–0.523, suggesting a robust association between miR-146a-5p and anthropometric characteristics of newborns, regardless of the glycemic status of their mothers. This negative correlation could be explained by the effect of OS/IFM associated with fetal growth, intrauterine fetal growth restriction (IUGR), premature labor, and amnion disorders on miR-146a-5p expression in tissues, which are major contributors to serum EV production. Our results are in line with the previous report on the upregulation of this microRNA in maternal blood during pregnancies with small-for-gestational-age fetuses and fetal growth restriction [78].

Apart from the upregulation in GDM vs. controls, miR-21-5p demonstrates a higher level of expression in the PBMCs of GDM patients with the associated pregnancy complications compared to those without detected adverse effects of GDM. The expression of this microRNA also correlates with anthropometric characteristics of neonates from GDM pregnancies. Therefore, both types of the evaluated parameters show a relation to miR-21-5p expression, which identifies this microRNA as a potentially valuable predictive indicator of pregnancy outcome in GDM. The association of the circulatory miR-21-5p expression with adverse pregnancy outcomes in GDM has been previously detected in two studies in the Chinese population [79,80], but for serum samples as biological specimens. Furthermore, an increased level of circulatory miR-21-5p has been associated with fetal macrosomia [80], which may be connected to our findings of a positive correlation with fetal growth and elevated expression in the adverse-outcome subgroup. The upregulation of miR-21-5p may also reflect a change in the expression in placental tissue, which is directly associated with the markers of fetal growth and macrosomia [44,81].

Our present pilot study provides the first evidence to support the diagnostic significance of mid-to-late pregnancy PBMC-derived microRNAs miR-146a-5p and miR-21-5p in GDM. Additionally, the detected upregulation of these microRNAs was confirmed in paired samples of serum-derived EVs, which strengthens the results of biomarker evaluation and also provides a means for the selection of an appropriate biological source of relevant biological markers. Since selected microRNAs participate in OS/IFM-related processes relevant to GDM and the associated complications, the detected correlations between the expression of microRNAs are consistent with the presence of common stimuli affecting the expression of these non-coding RNA molecules. Even though the capacity of these microRNAs as metabolic indicators proved to be limited, all three of them display a correlation between their expression levels and the values of redox status indicators, specifically NRF2 expression level and SOD activity. Importantly, we detected correlations between miR-146a-5p and miR-21-5p expression levels and neonatal characteristics, as well as the association of miR-21-5p upregulation with the occurrence of adverse pregnancy outcomes in GDM. However, there are certain limitations of the present study that should be acknowledged, including a small sample size, which restricts the statistical strength and could be a source of bias. Since this is a pilot study, during study design, we did not conduct a sample-size calculation necessary for reaching the preferable study power, due to the lack of previous results required for defining the statistical input data. When it comes to the potential occurrence of Type I errors, we acknowledge that corrections for multiple testing were not applied, and that some of the marginal weak correlations might have been lost after correcting the results. However, the relevance of microRNAs is supported by correlations with several different functionally related parameters, while the chances of false detection of microRNA dysregulation in GDM are reduced by matching findings for different types of samples. An inherent limitation is also the candidate-based design of our biomarker study, which, although justified, restricted the analysis to a small panel of microRNAs. In order to overcome the drawbacks, the main results require a carefully designed validation analysis in a much larger cohort recruited in a prospective manner. The association with pregnancy outcomes and relevance for OS/IFM status should be confirmed through a study regimen that includes several resampling events at different pregnancy stages, as well as the acquisition of information on dietary regimens and antioxidant intake. This would enable the assessment of fluctuations in microRNA levels throughout the pregnancy and evaluation of the optimal sampling period, as well as the estimation of the potential requirements for antioxidant interventions. The study design, which relies on the acquisition of samples between 24 and 30 weeks of gestation, does not allow assessment of the early diagnostic potential of selected microRNAs. Therefore, a prospective study that includes at least one sampling event during the early pregnancy stage would provide a means for the evaluation of miR-146a-5p and miR-21-5p from PBMCs and EVs as early diagnostic or predictive biomarkers, in terms of GDM onset. Regarding the potential of tested microRNAs as OS/IFM status indicators, our findings are correlative, and additional mechanistic studies with functional validation of supposed interactions are necessary for confirming the relation of OS/IFM stimuli with microRNA expression in relevant cell types and the regulatory roles of miR-146a-5p and miR-21-5p in OS/IFM-related pathways in GDM. Despite the acknowledged limitations, our novel data indicate the significance of OS/IFM-related microRNAs for GDM diagnosis and monitoring and the prediction of GDM-associated adverse pregnancy outcomes. Specifically, our findings demonstrate the potential of OS/IFM-related microRNAs miR-146a-5p and miR-21-5p to serve as indicators of GDM, associated OS-related changes, fetal growth, and pregnancy complications.

## 4. Materials and Methods

The patient recruitment procedure, inclusion and exclusion criteria for participant selection, and basic characteristics of the study group were previously described in detail in Stevanović et al. [46]. In brief, the GDM patient group comprised 50 pregnant women diagnosed between 24 and 28 weeks of gestation based on a 75 g oral glucose tolerance test (OGTT). The control group included an equal number of healthy, normoglycemic pregnant women matched for age and gestational stage to the GDM group. All potential participants provided written informed consent prior to the collection of relevant data and samples at the Gynecology and Obstetrics Clinic “Narodni Front” (OGC NF), Belgrade, Serbia. The Institutional Ethics Committee issued the approvals for the present research (No. 05006-2019-4925 from 18 March 2019 and No. 22008-2024-026903 from 18 December 2024). The research adhered to the ethical principles outlined in the Declaration of Helsinki.

Participants with a singleton pregnancy between 24 and 30 weeks of gestation were considered eligible for the study if they had available OGTT results and reported no history of autoimmune, metabolic, malignant, or psychiatric diseases or other pregnancy pathologies not related to GDM. After an overnight fasting period, peripheral blood samples were collected from participants in serum tubes and ethylenediaminetetraacetic acid (EDTA)-coated vacutainers.

A structured survey was designed to collect relevant clinical and epidemiological data, including age, gestational stage, anthropomorphic characteristics, personal and family anamnesis, comorbidities, therapies, dietary supplement intake, and smoking status. Comorbidities and the corresponding therapies were considered relevant for the exclusion of patients, according to the mentioned eligibility criteria. Participants consented to the collection of other relevant data from their medical records: hematological and biochemical parameters related to glycemic and lipid status (fasting glucose and insulin levels at sampling, OGTT results, levels of glycated hemoglobin (HbA1c), cholesterol, high-density lipoprotein (HDL), low-density lipoprotein (LDL), and triglycerides), kidney and liver function and inflammation status (CRP, fibrinogen, activities of alanine and aspartate transaminases (ALT and AST), serum albumin level and total serum proteins, creatinine, urea and uric acid, and sedimentation rate), concentration of metal ions and the corresponding binding proteins (iron, zinc, copper, hemoglobin, ferritin, transferrin, total iron-binding capacity—TIBC and alpha-2-macroglobulin—α2M, and complete blood cell count), data on GDM-related complications of pregnancy and delivery, as well as basic characteristics of the neonates (weight and length, Apgar score, and gestational age at birth). Adverse pregnancy outcomes were defined as spontaneous preterm labor, macrosomia, or IUGR, shoulder dystocia, and disorders of the amniotic fluid (oligohydramnios or polyhydramnios). Supplementary Table S1 presents an extended version of the patients’ data from Stevanović et al. [46] with additional characteristics.

### 4.1. Isolation of Peripheral Blood Mononuclear Cells

A detailed procedure for the isolation of PBMCs from EDTA-containing peripheral blood samples is detailed in Radojičić et al. [18]. In brief, buffy coats were obtained by centrifuging blood samples at 400× *g* (10 min at 4 °C) (Centrifuge 5804R, Eppendorf, Hamburg, Germany), collected by pipetting, diluted with phosphate-buffered saline (PBS, 10 mM, pH 7.4), and carefully layered onto lymphocyte separation medium (1.077 g/mL, LSM, Capricorn Scientific, Ebsdorfergrund, Germany). After a centrifugation step at 700× *g* (30 min at 18–21 °C), a PBMC layer was collected, then washed twice with PBS through centrifugation at 100× *g* for 10 min at room temperature and once with an erythrocyte lysis buffer (155 mM NH_4_Cl, 10 mM KHCO_3_, 0.1 mM EDTA, pH 7.2) following a 5 min incubation. The resulting PBMC pellet was thoroughly lysed in TRIzol reagent (Thermo Fisher Scientific, Waltham, MA, USA) and stored at −80 °C.

### 4.2. Isolation of EVs

Aliquots of serum samples (1 mL) stored at −80 °C were thawed on ice and used for the isolation of EVs by differential centrifugation. Contaminating cells, cellular debris, apoptotic bodies, and aggregates were removed by an initial centrifugation at 10,000× *g* (30 min at 4 °C) (Centrifuge 5804R, Eppendorf, Hamburg, Germany). The supernatant was carefully collected, diluted at a 1:9 ratio with cold sterile PBS (10 mM, pH 7.4, filtered through 0.1 μm pore-size filters (Sartorius, Göttingen, Germany)), and centrifuged at 100,000× *g* (60 min at 4 °C) (Optima L-90K ultracentrifuge, Beckman Coulter, Indianapolis, IN, USA). The supernatant was removed, while the pellet and the remaining ~1 mL of diluted serum were again diluted with PBS, and the centrifuging step was repeated (100,000× *g*, 60 min at 4 °C). Pelleted EVs were dissolved in TRIzol reagent (Thermo Fisher Scientific, Waltham, MA, USA) and stored at −80 °C for RNA extraction, while EV aliquots in PBS were used for the confirmation of EV presence and their characterization by dot-blot, Western blot, TEM, and NTA.

### 4.3. Western Blot and Dot Blot Analysis

CD63, NRF2, and GAPDH were detected by Western blot analysis, while the protein extraction procedure for both the EVs and PBMCs lysed in TRIzol (Thermo Fisher Scientific, Waltham, MA, USA) followed the protocol optimized by Stevanović et al. [82]. After electrophoretic separation on an 8% polyacrylamide gel under denaturing conditions and electroblotting of proteins onto nitrocellulose membrane (Cytiva, Marlborough, MA, USA), immunoblot analysis was performed using anti-CD63 (clone TS63, 1:1500) monoclonal antibody (Abcam, Cambridge, UK) for EV samples. The primary antibodies used for the immunoblot analysis of proteins isolated from PBMCs were the anti-NRF2 (1:1000) rabbit polyclonal antibody (Thermo Fisher Scientific, Waltham, MA, USA) and an anti-GAPDH (1:10,000) monoclonal antibody (Cell Signaling, Danvers, MA, USA). Sheep anti-rabbit IgG (AbD Serotec, Oxford, UK, 1:10,000) conjugated to horseradish peroxidase was used as the secondary antibody for the detection of NRF2 and GAPDH. In the case of CD63, the secondary antibody was biotinylated goat anti-mouse IgG (Vector Laboratories, Burlinghame, CA, USA, 1:5000), and the detection protocol included an incubation with Elite Vectastain ABC kit (Vector Laboratories, Burlinghame, CA, USA), according to the manufacturer’s instructions. Proteins were visualized by using Pierce ECL Western blotting substrate, according to the manufacturer’s instructions (Thermo Fisher Scientific, Waltham, MA, USA), and exposing a membrane to an X-ray film. The ChemiDoc MP Imaging System and Image Lab Version 6.1 software (Bio-Rad laboratories, Hercules, CA, USA) were employed for densitometric analysis.

For dot blot analysis, 3 µL of each serum-derived EV and a positive control (prostasome preparation, isolated according to Janković et al. [83]) were spotted onto a nitrocellulose membrane and allowed to dry. The membrane was blocked with 3% BSA in 50 mM PBS, pH 7.2, for 2 h at room temperature and subjected to immunodetection with an anti-CD63 antibody, following the same protocol as described above.

### 4.4. Transmission Electron Microscopy

The EV samples (10 µL) were applied to a carbon-coated grid by grid flotation for 45 min. Fixation was conducted using 2% formaldehyde for 10 min, followed by washing three times with 50 mM PBS, pH 7.2, for 2 min. The samples underwent post-fixation with 2% glutaraldehyde for 5 min, followed by a 5 min rinse in deionized water. After air-drying at room temperature, images were acquired using a CM12 electron microscope (Philips, Eindhoven, The Netherlands).

### 4.5. Nanoparticle Tracking Analysis (NTA)

A ZetaView Quatt PMX-430 (Particle Metrix, Inning am Ammersee, Germany) was employed for NTA in order to determine the concentration of isolated EVs and their size distribution, as previously described in Matijašević Joković et al. [84]. After an automatic cell check, the camera and laser were aligned, and the focus was validated using 100 nm polystyrene beads, following the manufacturer’s guidelines. The EV samples were diluted up to 1:1000 in sterile 50 mM PBS filtered through 0.1 μm pore-size filters, and the scattering mode measurements were conducted at 25 °C. The concentration calculation and size distribution analysis were performed using ZetaView software (Particle Metrix, Inning am Ammersee, Germany, version 8.05.16 SP3) with the following instrument settings: sensitivity—78%, shutter—100, 11 positions, and 2 cycles. Post-video capturing parameters were set to a minimal area of 10, a maximal area of 1000, and a minimum brightness of 30.

### 4.6. Isolation of Total RNA and Relative Quantification of microRNAs

PBMCs and serum-derived EVs lysed in TRIzol were used for the isolation of total RNA by phase separation and alcohol precipitation, following the protocol recommended by the manufacturer of the TRIzol reagent (Thermo Fisher Scientific, Waltham, MA, USA). Spectrophotometric analysis, based on absorbance at 260 and 280 nm (Epoch microplate spectrophotometer, BioTek Instruments–Agilent, Santa Clara, CA, USA), was employed for the quantification of the isolated RNA and for the assessment of RNA purity.

In order to preserve the integrity of the RNA during isolation, as well as to increase the RNA yield, the stored lysates were thawed on ice, and incubation steps were performed at −20 °C, while ice-cold isopropanol was used for the RNA precipitation with the addition of RNA-grade glycogen. The acquired RNA pellet was finally dissolved in diethyl pyrocarbonate (DEPC)-water and stored at −80 °C.

The potential DNA contamination of the extracted RNA was eliminated by DNase treatment (Amplification grade DNase I, Sigma-Aldrich, Burlington, MA, USA), performed according to the manufacturer’s protocol and immediately followed by reverse transcription (RT). Apart from the DNase-treated RNA, the RT reaction mixture included 100 U of RevertAid Reverse Transcriptase (Thermo Fisher Scientific, Waltham, MA, USA) with the recommended volume of the corresponding reaction buffer, 250 nM of stem-loop primers (Microsynth, Balgach, Switzerland) (Table 3), and 0.5 mM of dNTPs, in a total volume of 20 µL. The following temperature profile was used for RT: 30 min incubation at 16 °C, 30 min at 42 °C, and 5 min at 85 °C. The acquired cDNA was diluted with DNase-free water (at least 5×) for preparing qPCR reaction mixtures that contained SYBR™ Green PCR Master Mix (Thermo Fisher Scientific, Waltham, MA, USA), forward and reverse primers (Microsynth, Balgach, Switzerland) (Table 3) in equimolar concentrations (0.625 µM for miR-191-5p and miR-146a-5p, 0.375 µM for miR-155-5p and miR-21-5p), and DNase-free water. Applied Biosystems 7500 Real-Time PCR System (Thermo Fisher Scientific, Waltham, MA, USA) was programmed to the following temperature profile for qPCR reactions: 10 min at 95 °C for the initial denaturation and enzyme activation, and 40 cycles that included denaturation for 15 s at 95 °C and primer annealing with elongation for 1 min at 60 °C. The results of the qPCR were analyzed using Applied Biosystems 7500 Software v2.3 (Thermo Fisher Scientific, Waltham, MA, USA). A ubiquitous and consistently expressed microRNA, miR-191-5p (miRBase: MIMAT0000440), was used as an internal reference for both the PBMC and EV samples. The relative expression of miR-146a-5p (miRBase: MIMAT0000449), miR-155-5p (miRBase: MIMAT0000646), and miR-21-5p (miRBase: MIMAT0000076) was evaluated by the delta delta Ct (∆∆Ct) method, while fold changes between the GDM group and controls were calculated based on the 2^−∆∆Ct^ values. The expression of miR-155-5p in the EVs was not evaluated due to poor qPCR signal and inconsistencies in the acquired results of relative quantification.

### 4.7. Activities of Antioxidant Enzymes

The detailed procedure for measuring the activities of the glutathione reductase (GR), superoxide dismutase (SOD), and catalase (CAT) has been described in Stevanović et al. [46]. In brief, hemolysates stored at −80 °C were used for assessing enzyme activities, while all measurements were conducted in triplicate. The GR activity was measured according to Glatzle et al. [46,85] by spectrophotometric analysis (Epoch microplate spectrophotometer, BioTek Instruments-Agilent, Santa Clara, CA, USA). The oxidation of NADPH to NADP^+^ was monitored in 96-well plate format, in a reaction mixture containing 60 µL of potassium phosphate buffer (KB, 100 mM with 1 mM EDTA, pH 7.5), 10 µL of NADPH (1 mM), 10 µL of oxidized glutathione (2 mM), 10 µL of EDTA (0.5 M), 180 µL of distilled water, and 20 µL of the diluted hemolysate (10× in KB). The absorbance was recorded at 340 nm for 150 s, and the GR activity was calculated by using the extinction coefficient of NADPH.

The SOD activity was measured according to Beauchamp and Fridovich [46,86], in an assay based on the inhibition of nitroblue tetrazolium (NBT) reduction. Briefly, reaction mixtures containing 175 µL of KB, 10 µL of methionine (13 µM), 8 µL of NBT (75 µM), 6 µL of hemolysate, and 1 µL of riboflavin (2 µM) were spectrophotometrically analyzed in a 96-well plate format, after an incubation period of 30 min under UV light. The absorbance was measured at 560 nm, and ΔA (Ablank − Asample) represented the inhibitory activity of SOD, while one IU was assessed as 50% of the inhibition.

CAT activity was measured according to the method described by Claiborn [46,87] by measuring absorbance at 240 nm for 60 s. A reaction mixture containing 202.5 µL of KB, 22.5 µL of H_2_O_2_ (50 mM), and 22.5 µL of the hemolysate was prepared in a quartz cuvette, followed by an immediate recording of the absorbance at 240 nm. CAT activity was calculated by using the extinction coefficient of H_2_O_2_.

### 4.8. Oxidative Modifications of Biomacromolecules

Ellman’s method was used for assessing the serum thiol content in a 96-well plate format via spectrophotometric analysis. Reaction mixtures were prepared by mixing serum samples (30 µL, stored at −80 °C) with equal volumes of 2 mM 5,5′-dithiobis(2-nitrobenzoic acid) (DTNB) and 1 M Tris buffer (pH 8.0). The volume was made up to 300 µL with distilled water, and the absorbance at 412 nm was recorded after a 30 min incubation period. The serum thiol content was calculated using the extinction coefficient of the reagent.

Serum samples were subjected to the assessment of total protein carbonyl (PCO) concentration after protein derivatization using 2,4-dinitrophenylhydrazine (DNPH), according to a commonly used method described by Levine et al. [46,88]. Serum samples were diluted with distilled water (to 10 mg/mL protein), and a volume of 500 µL was mixed with 250 µL of 10% trichloroacetic acid (TCA). After a centrifugation step of 5 min at 1500× *g*, the precipitated proteins were incubated with 250 µL of DNPH (10 mM) in 2 M HCl for 30 min at 25 °C, with periodic vigorous mixing. After precipitating proteins with 500 µL of TCA (10%), centrifuging was repeated, and the pellet was washed twice with 1 mL of ethanol-ethyl acetate (1:1 ratio), with thorough mixing. A 1.5 mL volume of 2% sodium dodecyl sulphate (SDS) prepared in phosphate buffer (0.08 M, pH 8.0) with 0.05% EDTA was used to dissolve the final precipitate during incubation at 37 °C for 20 min. The absorbance was measured at 375 nm, and the concentration of PCO was calculated using the extinction coefficient (22,000 M^−1^ cm^−1^) and expressed as nmol/mg of protein.

The serum content of TBARSs was also measured spectrophotometrically. Undiluted serum (0.1 mL) was mixed with 0.2 mL of TBARS reagent (15% TCA, 2% HCl, 0.375% 2-thiobarbituric acid), heated in a boiling water bath for 15 min, cooled, and centrifuged at 3000× *g* for 10 min. The absorbance of the supernatant was measured at 532 nm against a blank in a 96-well plate format (Epoch microplate spectrophotometer, BioTek Instruments—Agilent, Santa Clara, CA, USA). TBARS content was calculated using the extinction coefficient (156,000 M^−1^ cm^−1^) and expressed as μmol/L.

### 4.9. Statistical Analysis

RStudio (RStudio Team, Boston, MA, USA) software v. 4.4.1 was used for the statistical analyses of the acquired data, as well as for constructing box plots and scatter charts. Continuous variables were assessed for normality of distribution using the Kolmogorov–Smirnov test, while differences in variance between groups were evaluated by the F-test. A two-tailed Student’s t-test was employed for comparing normally distributed data, while results with significant deviations from normal distribution were compared by the Mann–Whitney U test. Categorical variables were evaluated by the χ^2^ test. Correlation tests relied on linear regression, reporting the Pearson correlation coefficient (r) and the corresponding *p*-values. A *p*-value of 0.05 was considered a threshold for statistical significance. Results of statistical analysis were not adjusted for potential confounders, including dietary supplement intake, which varied significantly between eligible participants (dosages, regimen, and formulations).

## Figures and Tables

**Table 1:** Correlations of the expression of miR-146a-5p, miR-155-5p, and miR-21-5p (−ΔCt) with the values (log2n) of lipid and glycemic status indicators in GDM patients.

Sample Type		miR-146a-5p		miR-155-5p		miR-21-5p	
		r	*p* Value	r	*p* Value	r	*p* Value
**PBMCs**	Lipid profile						
	Triglycerides	−0.131	0.427	−0.226	0.166	−0.061	0.712
	Cholesterol	−0.100	0.550	−0.010	0.952	0.236	0.154
	HDL	0.065	0.664	0.154	0.301	0.091	0.548
	LDL	0.085	0.612	0.039	0.816	0.194	0.243
	Glycemic profile						
	Fasting glucose	−0.037	0.805	0.114	0.445	0.018	0.905
	OGTT 60′	−0.144	0.334	−0.012	0.936	0.062	0.682
	OGTT 120′	−0.094	0.530	0.036	0.810	−0.049	0.746
	Fasting insulin	0.230	0.120	0.137	0.358	0.164	0.276
	HOMA-IR	0.230	0.138	0.137	0.381	0.130	0.412
	HOMA-β	0.134	0.392	0.114	0.447	0.224	0.154
	HbA1c	0.064	0.756	0.264	0.192	−0.182	0.384
**EVs**	Lipid profile						
	Triglycerides	0.166	0.306	-	-	0.298	0.065
	Cholesterol	0.051	0.755	-	-	0.241	0.139
	HDL	0.082	0.575	-	-	−0.063	0.674
	LDL	0.007	0.956	-	-	0.173	0.292
	Glycemic profile						
	Fasting glucose	0.057	0.697	-	-	0.309	0.034 *
	OGTT 60′	−0.023	0.875	-	-	0.139	0.351
	OGTT 120′	0.178	0.221	-	-	0.352	**0.015**
	Fasting insulin	−0.144	0.324	-	-	0.236	0.110
	HOMA-IR	−0.116	0.453	-	-	0.282	0.070
	HOMA-β	−0.183	0.234	-	-	0.111	0.484
	HbA1c	−0.137	0.496	-	-	0.103	0.624

* Statistically significant results are shown in bold. Abbreviations: GDM—gestational diabetes mellitus; OGTT—oral glucose tolerance test; HOMA-IR—homeostatic model assessment of insulin resistance; HOMA-β—homeostatic model assessment of β-cell function; HbA1c—glycated hemoglobin; HDL—high-density lipoprotein; LDL—low-density lipoprotein; r—Pearson’s correlation coefficient.

**Table 2:** Correlations of the expression of miR-146a-5p, miR-155-5p, and miR-21-5p (−dCt) with the values (log2n) of indicators of antioxidant response and redox status in GDM patients.

Sample Type		miR-146a-5p		miR-155-5p		miR-21-5p	
		r	*p* Value	r	*p* Value	r	*p* Value
**PBMCs**							
	NRF2 mRNA	0.096	0.521	−0.059	0.521	0.183	0.223
	NRF2 protein	0.381	0.054	**0.401**	0.042 *	**0.396**	**0.050**
	GR (IU/mg Hgb)	0.026	0.873	0.080	0.624	0.133	0.420
	SOD (IU/mg Hgb)	0.264	0.109	−0.122	0.466	**0.361**	**0.024**
	CAT (IU/mg Hgb)	0.012	0.941	0.184	0.256	−0.004	0.981
	SH (mmol/g prot.)	0.159	0.347	−0.017	0.920	−0.052	0.763
	PCO (mmol/g prot.)	0.057	0.791	0.224	0.293	−0.111	0.614
	TBARS (µmol/L)	0.026	0.864	−0.064	0.673	0.037	0.809
**EVs**							
	NRF2 mRNA	**−0.357**	**0.012**	-	-	−0.132	0.376
	NRF2 protein	0.090	0.662	-	-	0.049	0.820
	GR (IU/mg Hgb)	−0.139	0.399	-	-	−0.069	0.681
	SOD (IU/mg Hgb)	**−0.400**	**0.011**	-	-	−0.204	0.219
	CAT (IU/mg Hgb)	−0.279	0.077	-	-	−0.066	0.686
	SH (mmol/g prot.)	0.279	0.090	-	-	0.211	0.210
	PCO (mmol/g prot.)	−0.082	0.690	-	-	0.049	0.816
	TBARS (µmol/L)	−0.238	0.107	-	-	0.059	0.700

* Statistically significant results are shown in bold. Abbreviations: PBMCs—peripheral blood mononuclear cells; EVs—extracellular vesicles; GR—glutathione reductase; SOD—superoxide dismutase; CAT—catalase; SH—sulfhydryl (thiol) group; PCO—protein carbonyl; TBARS—thiobarbituric acid reactive substances; r—Pearson’s correlation coefficient.

**Table 3:** Primer sequences.

	Primer	Sequence
RT-PCR	miR-191-5p-RT	5′-GTCGTATCCAGTGCAGGGTCCGAGGTATTCGCACTGGATACGACCAGCTG-3′
	miR-146a-5p-RT	5′-GTCGTATCCAGTGCAGGGTCCGAGGTATTCGCACTGGATACGACAACCCA-3′
	miR-155-5p-RT	5′-GTCGTATCCAGTGCAGGGTCCGAGGTATTCGCACTGGATACGACAACCCC-3′
	miR-21-5p-RT	5′-GTCGTATCCAGTGCAGGGTCCGAGGTATTCGCACTGGATACGACTCAACAT-3′
qPCR	Universal miR-rv	5′-CCAGTGCAGGGTCCGAGGTAT-3′
	miR-191-5p-fw	5′-CGCGGTCAACGGAATCCCAAAAG-3′
	miR-146a-5p-fw	5′-CGGCGGTTGAGAACTGAATTCCA-3′
	miR-155-5p-fw	5′-GGCGTTAATGCTAATCGTGATAG-3′

## Data Availability

The raw data supporting the conclusions of this article will be made available by the authors on reasonable request.

## Supplementary Materials

The following supporting information can be downloaded at https://www.mdpi.com/article/10.3390/ijms26146902/s1.

## Abbreviations

The following abbreviations are used in this manuscript:

OS	Oxidative stress
IFM	Inflammation
GDM	Gestational diabetes
PBMCs	Peripheral blood mononuclear cells
EVs	Extracellular vesicles
IDF	International Diabetes Federation
T2DM	Type 2 diabetes
ROS	Reactive oxygen species
OGTT	Oral glucose tolerance test
OGC NF	Gynecology and Obstetrics Clinic “Narodni Front”
HbA1c	Glycated hemoglobin
HDL	High-density lipoprotein
LDL	Low-density lipoprotein
ALT	Alanine aminotransferase
AST	Aspartate aminotransferase
TIBC	Total iron-binding capacity
α2M	Alpha-2-macroglobulin
IUGR	Intrauterine fetal growth restriction
PBS	Phosphate-buffered saline
LSM	Lymphocyte separation medium
TEM	Transmission electron microscopy
NTA	Nanoparticle tracking analysis
GR	Glutathione reductase
SOD	Superoxide dismutase
CAT	Catalase
KB	Potassium phosphate buffer
DTNB	5,5’-dithiobis(2-nitrobenzoic acid)
PCO	Protein carbonyl
DNPH	2,4-dinitrophenylhydrazine
TCA	Trichloroacetic acid
SDS	Sodium dodecyl sulphate
TBARSs	Thiobarbituric acid-reactive substances
MCV	Mean corpuscular volume
MCH	Mean corpuscular hemoglobin
CRP	C-reactive protein
TIBC	Total iron binding capacity
T1DM	type 1 diabetes mellitus
HUVECs	human umbilical vein endothelial cells
EDTA	ethylenediaminetetraacetic acid
DEPC	diethyl pyrocarbonate
