# Supplementary material for: Upregulation of the Antioxidant Response-Related microRNAs miR-146a-5p and miR-21-5p in Gestational Diabetes: An Analysis of Matched Samples of Extracellular Vesicles and PBMCs
Source: Int J Mol Sci. 2025 Jul 18;26(14):6902. doi: 10.3390/ijms26146902 (PMC12295923; doi:10.3390/ijms26146902)
Supplement: Supplementary file 1 [file ijms-26-06902-s001.zip › ijms-3699745-supplementary.pdf]

Supplementary Table S1. Basic characteristics of study participants.

	GDM patients	Controls	*P* value
N	50	50	
Age (years) [a]	35.2±4.2	33.4±4.7	0.051
Gestational age at sampling (weeks) [a]	27.2±2.1	26.5±1.6	0.056
Smoking status (%)	20	24	0.23
GDM in previous pregnancies (%)	4 (8)	-	
Family history of diabetes (%)	38	20	**0.047***
Gravidity, n (%)			
1	17 (34)	20 (40)	0.42
2	18 (36)	12 (24)	
≥3	15 (30)	18 (36)	
Parity, n (%)			
0	25 (50)	28 (56)	0.83
1	18 (36)	16 (32)	
≥2	7 (14)	6 (12)	
Pre-pregnancy weight (kg) [a]	70.8±13.0	66.4±8.1	**0.049**
Weight at sampling (kg) [a]	77.8±12.2	74.4±9.2	0.13
Height (cm) [a]	169.0±5.1	170.8±6.6	0.14
Weight gain (kg) [a]	6.8±5.0	6.3±11.3	0.79
Pre-pregnancy BMI	24.8±4.7	22.8±2.5	**0.009**
Glycaemic status [a]			
OGTT (mmol/L)			
0'	4.89±0.66	4.42±0.40	**4.24e-5**
60'	10.84±1.49	7.73±1.21	**3.23e-19**
120'	9.25±1.93	6.6±0.98	**4.15e-12**
Fasting insulin (mU/L)	18.07±15.85	18.67±20.34	0.56
HOMA-IR	4.29±4.71	3.26±2.38	0.16
HOMA-β	61.94±43.84	75.38±45.08	0.07
HbA1c (%)	4.78±0.22	4.67±0.23	0.09
Lipid profile [a]			
Triglycerides (TG) (mmol/L)	2.38±0.79	2.15±0.72	0.20
Cholesterol (mmol/L)	6.72±1.14	6.86±1.21	0.60
HDL (mmol/L)	1.85±0.41	2.17±0.50	**0.001**
LDL (mmol/L)	3.82±1.03	3.71±1.14	0.66
TG/HDL	1.40±0.61	1.14±0.64	0.09
LDL/HDL	2.16±0.69	1.76±0.65	**0.017**
Other biochemical parameters [a]			
Total proteins (g/L)	64.12±3.42	64.31±4.67	0.83
Albumin (g/L)	34.50±2.37	35.97±2.98	**0.021**
Urea (mmol/L)	3.17±1.02	2.98±0.78	0.35
Creatinine (µmol/L)	50.81±7.09	51.80±6.75	0.52
Uric acid (µmol/L)	239.76±52.48	215.20±38.16	**0.019**
CRP (mg/L)	6.80±8.45	6.06±4.24	0.61
AST (U/L)	17.50±6.46	18.15±5.26	0.67
ALT (U/L)	19.83±10.47	16.06±8.75	0.06
Bilirubin (µmol/L)	2.38±0.57	2.80±1.49	0.10
Fibrinogen (g/L)	3.88±0.88	3.40±0.71	**0.047**
Copper (µmol/L)	25.79±8.81	24.56±7.82	0.49
Zinc (µmol/L)	11.27±2.44	12.45±2.94	**0.042**
Iron (µmol/L)	15.36±5.85	16.51±8.04	0.48

Ferritin (μg/L)	20.48±14.34	16.49±13.06	0.11
TIBC (μmol/L)	59.96±11.30	60.07±10.08	0.96
Transferrin (g/L)	2.90±0.31	2.92±0.25	0.78
Complete blood count [a]			
Erythrocytes (10^{12} cells/L)	3.80±0.30	3.69±0.36	0.14
Haemoglobin (g/L)	116.40±7.95	110.25±9.04	**0.0018**
Haematocrit	0.344±0.025	0.329±0.027	**0.012**
Sedimentation rate (mm/h)	34.55±15.77	33.38±12.50	0.72
MCV (fL)	90.62±3.96	89.67±6.07	0.41
MCH (pg/cell)	30.70±1.59	29.98±2.07	0.087
MCHC (g/L)	338.31±9.15	335.22±8.26	0.12
Leucocytes (10^9 cells/L)	9.75±2.15	9.62±1.94	0.78
Thrombocytes (10^9 cells/L)	239.93±60.82	237.42±45.95	0.84
Granulocytes (10^9 cells/L)	6.98±1.84	6.30±2.20	0.14
Lymphocytes (10^9 cells/L)	2.11±0.41	3.14±3.77	0.51
Newborn characteristics and obstetric complications [a]			
Weight (g)	3473.5±480.3	3602.3±457.5	0.26
Length (cm)	51.20±2.44	51.39±4.04	0.79
BMI	12.90±0.95	13.15±1.00	0.30
Apgar score at 1 min	8.88±0.50	8.68±1.00	0.28
Preterm labor (%)	17	7.9	0.22
Macrosomia (%)	14.6	18.4	0.65
Polyhydramnios (%)	9.8	7.9	0.77

[a] Mean±SD,
*Statistically significant results are shown in bold

Abbreviations: GDM - gestational diabetes mellitus; BMI - body mass index; OGTT - oral glucose tolerance test; HOMA-IR - homeostatic model assessment of insulin resistance; HOMA-β - homeostatic model assessment of β cell function; HbA1c – glycated haemoglobin; TG – triglycerides; HDL - high-density lipoprotein; LDL - low-density lipoprotein; CRP – C-reactive protein; AST – aspartate aminotransferase; ALT – alanine aminotransferase; TIBC – total iron-binding capacity; MCV – mean corpuscular volume; MCH – mean corpuscular haemoglobin; MCHC – mean corpuscular haemoglobin concentration.
